# Paradoxical Psoriasis Induced by Ustekinumab: A Comprehensive Review and Case Report

**DOI:** 10.3390/medicina60010106

**Published:** 2024-01-06

**Authors:** Andrei Ovidiu Olteanu, Artsiom Klimko, Ioana Tieranu, Olguta Anca Orzan, Cristian Valentin Toma, Elena Mirela Ionescu, Carmen Monica Preda, Cristian George Tieranu

**Affiliations:** 1 Department of Gastroenterology, “Carol Davila” University of Medicine and Pharmacy, 020021 Bucharest, Romania; ovidiu-andrei.olteanu@drd.umfcd.ro (A.O.O.); mirela.ionescu@umfcd.ro (E.M.I.); carmen.preda@umfcd.ro (C.M.P.); cristian.tieranu@umfcd.ro (C.G.T.); 2 Department of Gastroenterology, Elias Emergency University Hospital, 011461 Bucharest, Romania; 3 Laboratory of Molecular Neuro-Oncology, Department of Neurology, University Hospital Zurich, 8091 Zürich, Switzerland; artsiom.klimko@usz.ch; 4 Department of Pediatrics, “Maria Sklodowska Curie” Clinical Emergency Hospital for Children, 077120 Bucharest, Romania; dr.cindeaioana@yahoo.ro; 5 Department of Dermatology, “Carol Davila” University of Medicine and Pharmacy, 020021 Bucharest, Romania; 6 Department of Dermatology, Elias Emergency University Hospital, 011461 Bucharest, Romania; 7 Department of Innovation and e-Health, “Carol Davila” University of Medicine and Pharmacy, 020021 Bucharest, Romania; cristian.toma@umfcd.ro; 8 Department of Gastroenterology, Fundeni Clinical Institute, 022328 Bucharest, Romania

**Keywords:** ustekinumab, paradoxical psoriasis, biologics, Crohn’s disease

## Abstract

Ustekinumab (UST), a biologic agent targeting interleukin-12 and interleukin-23, is widely used in the management of psoriasis and Crohn’s disease. Despite its efficacy, there have been instances of paradoxical psoriasis induction or exacerbation in some patients during UST therapy. This paper offers a comprehensive review of reported cases of UST-induced paradoxical psoriasis, including a case from our clinic. We focus on a 39-year-old female patient with a history of long-standing Crohn’s disease who developed a psoriasiform rash, as confirmed by biopsy, while undergoing UST treatment. The patient’s clinical journey, from initial diagnosis through the complexities of treatment adjustments due to various complications including drug-induced lupus and the subsequent onset of psoriatic manifestations, provides insight into the challenges encountered in the clinical management of such cases. This review emphasizes the necessity for clinicians to recognize the possibility of paradoxical psoriasis in patients receiving UST treatment and calls for further research to better understand this phenomenon and devise effective management strategies.

## 1. Introduction

Crohn’s disease (CD) is a chronic immune-mediated disorder characterized by fluctuating clinical activity and periods of remission [1]. The treatment of CD has evolved from immunomodulatory drugs to include, more recently, biologic agents, which represent the mainstay of therapy in the last decade. These advancements allow clinicians to aim for deeper remission and improved long-term outcomes [2,3]. Ustekinumab (UST) has recently received Food and Drug Administration and European Medicines Agency approval for CD and ulcerative colitis (UC) treatment [4]. By targeting the interleukin (IL) 12/23 pathway, UST offers a new treatment option alongside tumor necrosis factor (TNF)-antagonists.

Apart from its proven efficacy in treating CD and UC, UST has also shown a favorable safety profile in a recent analysis from pooled phase 2 and phase 3 studies on inflammatory bowel disease patients after 1 year of follow-up [5]. Indeed, these results come as confirmation to a previously reported safety profile from the Psoriasis Longitudinal Assessment and Registry (PSOLAR) which focused on malignancy, major adverse cardiovascular events, infections, and mortality over more than 12,000 UST patient-years of follow-up [6].

However, UST treatment has been associated with a paradoxical phenomenon in some patients, where it induces or exacerbates psoriasis, a condition it is ordinarily used to treat.

Psoriasis is a chronic, inflammatory skin disease that affects around 2–3% of people worldwide and significantly impacts the quality of life of those affected [7]. It may exhibit various clinical manifestations with chronic plaque psoriasis being the most common subtype of the disease. Chronic plaque psoriasis is characterized by well-defined erythematous plaques covered by thick silver scales. The psoriatic lesions can be localized and are usually symmetrically distributed, but they can also be generalized. The most frequently affected areas are the extensor parts of the body such as knees and elbows, as well as the scalp, palms, and soles. When the lesions involve the palms and soles, painful fissures may also be seen [7]. Its pathophysiology involves immune dysregulation, where antigens activate innate immune cells in the skin, leading to the secretion of pro-inflammatory cytokines, namely, IL-12, IL-17a, and IL-23. These cytokines drive a positive feedback loop of keratinocyte proliferation and further inflammation [8]. IL-12 and IL-23 are central to the CD4+ T-cell response (normally regulating the Th1 and Th17 responses, respectively)—both cytokines are dimeric and share the p40 subunit, which can be targeted by the monoclonal antibody UST to treat psoriasis [9].

Paradoxical psoriasis (PP) represents a non-infectious, immune-mediated side effect with different biological agents. Albeit sharing clinical manifestations with the classical form of psoriasis, their immunological pathways are rather divergent [10]. The immunological hallmark of PP is the over-production of interpheron-α (IFN-α) secondary to TNF-α inhibition [11]. Delving deeper into PP pathophysiology, it shares similar features with early-stage classical idiopathic psoriasis, mostly based upon innate immunity components like plasmacytoid dendritic cells (pDCs), neutrophils, macrophages, mast cells, and monocytes [12]. An increased number of pDCs in PP skin samples suggests a pDC-driven IFN-α overexpression immunological pathway [13]. A notable difference from classical idiopathic psoriasis is the lack of T cell activation [11].

PP acts like a multifactorial disease with several single-nucleotide polymorphisms (SNPs) being linked to the development of psoriasiform skin lesions in patients treated with anti-TNF-α agents. Additional risk factors include a family history of psoriasis, active smoking, and psychological stressors. These risk factors, associated with genetic susceptibility, generate and perpetuate an interesting pathological interplay [14].

Tackling the aforementioned cascade of events for the management of PP involves simultaneously treating the underlying disorder and the skin lesions. The medical attitude in front of PP should be based on the balance between the underlying disease control and the cutaneous side effects of the treatment. For instance, patients with attained disease control and mild-to-moderate skin reactions should continue current therapy, and skin reactions should be treated with added topical agents such as steroids, vitamin D, or calcineurin inhibitors, while in severe cutaneous lesions, systemic steroids and other immunosuppressive agents should be added. It is critical to note that stopping biological treatment for the underlying disease is not prerequisite in this scenario [15]. In contrast, where there is a lack of clinical control of the underlying disease with the current treatment apart from cutaneous side effects, it is obvious that the suspension or replacement of the causative agents is advisable [16].

Taking into consideration the above-mentioned data, PP presents a unique challenge to clinicians. Despite its clinical relevance, paradoxical psoriasis remains under-researched and poorly understood.

This paper aims to provide a comprehensive review of the reported cases of UST-induced paradoxical psoriasis, including a case from our clinic.

## 2. Case Report

We report the case of a 39-year-old female patient with a long-standing history of CD, who developed a biopsy-confirmed psoriasiform rash while undergoing treatment with UST. Initially diagnosed with colonic CD in 2007, she was treated with infliximab (IFX), achieving clinical remission through standard dosing induction and maintenance regimens. This treatment was successfully continued until 2014 when she developed antibody-confirmed drug-induced lupus [17]. Subsequently, based on a rheumatologist’s recommendation, her treatment was switched to adalimumab (ADA). ADA maintained the clinical remission of CD and resolved the lupus rash and associated arthropathy. In 2018, she experienced a moderate flare of CD, confirmed clinically and endoscopically, necessitating a dosage increase of ADA to regain clinical remission. This remission lasted until 2020, when another moderate flare-up occurred, as indicated by the Crohn’s Disease Activity Index (CDAI). It was then decided to switch to UST, administered as a standard weight-based intravenous dose of 390 mg. Clinical remission was achieved post induction and sustained for three years with UST 90 mg subcutaneously every 12 weeks until 2023, with no significant episodes affecting her CD.

**Figure 1:**
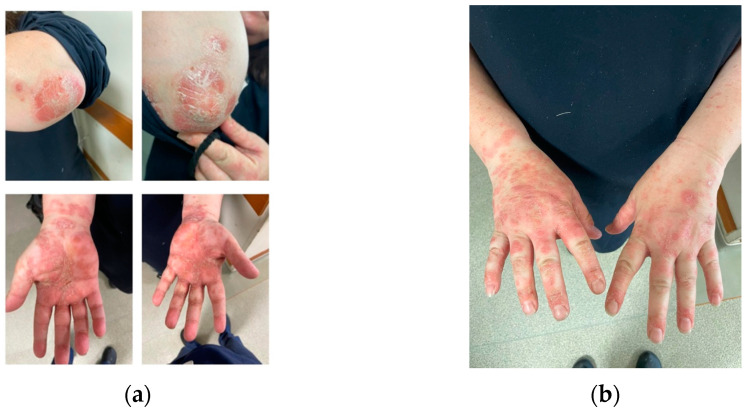
Squamous lesions involving the elbows and the palmar aspect of the hands (**a**), as well as the dorsal side of the hands in a CD patient treated with UST (**b**).

In June 2023, the patient presented to our department with a squamous rash on her hands, elbows, and scalp, as shown in Figure 1a,b.

**Figure 2:**
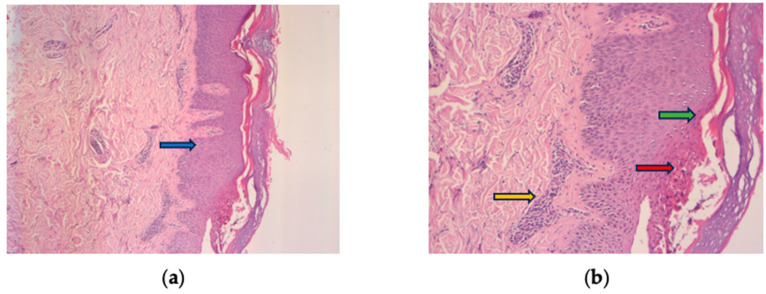
Histologic findings typically of psoriasis: epidermal hyperplasia (blue arrow), parakeratosis (green arrow), Munro’s micro abscesses (red arrow), thinned granular cell layer of the epidermis, dilated dermal capillaries, and inflammatory infiltrate in the upper dermis (yellow arrow). (**a**) HE 10× and (**b**) HE 30× magnification.

**Figure 3:**
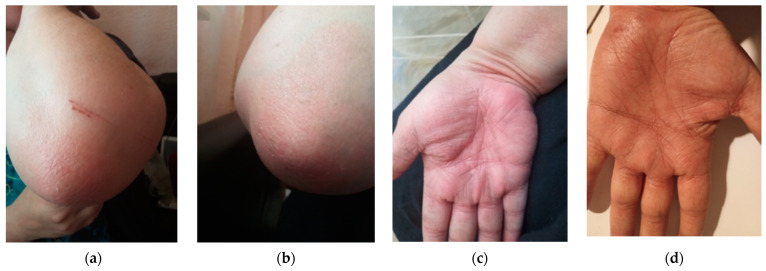
Complete resolution of squamous lesions after 4 weeks of treatment with topical steroids without UST discontinuation (**a**) left elbow (**b**) right elbow (**c**) right palm (**d**) left palm.

A skin biopsy was performed as recommended by the Dermatology consultation and confirmed the diagnosis by revealing epidermal hyperplasia, parakeratosis, Munro’s micro abscesses, thinned granular cell layer of the epidermis, dilated dermal capillaries, and dermal inflammatory infiltrate (Figure 2a,b). Considering the improvement in her difficult-to-treat CD, the decision was made to continue UST treatment and introduce topical treatment with beclomethasone ointment for the psoriatic lesions. This therapeutic approach, although empirical, addressed a significant gap in current knowledge. The patient’s re-evaluation after four weeks of topical steroid treatment showed a complete resolution of the squamous lesions without discontinuing UST (Figure 3a–d).

## 3. Literature Review

To identify patient characteristics, management strategies, and clinical outcomes associated with UST-induced paradoxical psoriasis, we conducted a comprehensive literature search in the PubMed and Scopus databases. Specific keywords and Medical Subject Heading (MeSH) terms pertinent to UST and paradoxical psoriasis were employed for accuracy. These terms included ‘ustekinumab’, ‘paradoxical psoriasis’, ‘biologic-induced psoriasis’, and ‘adverse reactions’. Inclusion criteria were (1) peer-reviewed case-report articles, case-series or randomized controlled trials; (2) patients diagnosed with paradoxical psoriasis while on UST therapy; (3) availability of detailed clinical data; and (4) English-language publications. Exclusion criteria encompassed review articles and letters without original data. To broaden our search and capture as many reported cases as possible, we conducted a thorough review of the references in the cited case-reports. Additionally, we explored online abstract books covering the three major gastroenterology congresses—Digestive Disease Week, United European Gastroenterology Week, and European Crohn’s and Colitis Organization. This step ensured we did not overlook any abstracts that our initial search strategy might have missed. Our efforts yielded ten cases of patients with UST-induced paradoxical psoriasis. A comprehensive summary of our findings is presented in Table 1.

In our review, the patients’ ages ranged from 24 to 58 years, with a slight female predominance. Most of the patients were of Caucasian ethnicity, with one case describing an Asian patient (Patient #4). The onset of paradoxical psoriasis post UST initiation varied widely, from as early as two weeks to as late as three years. While the dosing regimens of UST exhibited differences across cases, a consistent finding was the onset of paradoxical psoriasis subsequent to its administration. Clinical manifestations varied, including palmoplantar pustular, subcorneal pustular, classical plaque, and pustular morphologies, primarily affecting the hands, trunk, face, limbs, and scalp. Notably, Wenk et al. and Hay et al. observed a change in psoriasis morphology from plaque to pustular form post UST initiation [23,25].

In terms of management, most patients had their UST treatment discontinued and were switched to another biologic agent, such as golimumab or adalimumab. However, there were notable exceptions where the patient continued UST and managed the rash with topical corticosteroids or intensified UST therapy. Caca-Biljanovska et al. reported successful psoriatic flare management with UST dose escalation, drawing on findings from the PHEONIX-2 trial that linked dose intensification to increased efficacy [24,28]. The authors switched the patient to an intensified dose regimen every 8 weeks, instead of the recommended 12—by week 28. Following psoriasis flare clearance, the patient was transitioned back to the standard 12-week protocol.

Concurrent gastrointestinal conditions, such as Crohn’s disease, were common among the patients. Comorbidities varied and included conditions such as asthma, corticosteroid-induced osteoporosis, type II diabetes, hypertension, hypercholesterolemia, and various forms of arthritis. Before UST treatment, patients were commonly treated with a variety of therapies, including systemic corticosteroids, mesalazine, azathioprine, adalimumab, infliximab, methotrexate, and various forms of phototherapy. The time to resolution of the paradoxical psoriasis after the discontinuation of UST or switching to another agent was typically within a few weeks, although the exact time frame was not consistently reported across the studies. Follow-up durations ranged from 18 months to not specified.

## 4. Discussion

The phenomenon of paradoxical psoriasis induced by UST, as observed in the cases reviewed, highlights the complexity of psoriasis as a disease and the intricate role of the immune system in its pathogenesis. Therapeutic monoclonal antibodies, which target TNF-α (adalimumab, etanercept, and infliximab) or the p40 subunit of IL-12 and IL-23 (e.g., UST), have become pivotal in managing severe psoriasis and are generally well tolerated. However, paradoxical cutaneous reactions are rare but notable. Murphy et al. identified 2043 cases, with the majority (91.2%) being caused by TNF-α inhibitors and only a small fraction (2.4%) by p40 inhibitors [16]. The mechanisms behind these paradoxical reactions are not well understood and may vary across drug classes. It is hypothesized that the selective inhibition of specific cytokines may inadvertently disrupt the immune balance, leading to the onset or worsening of psoriasis.

While the selective inhibition of specific cytokines can certainly contribute to the emergence of paradoxical psoriasis, recent insights from pharmacogenetic studies suggest an even deeper layer of complexity. Single-nucleotide polymorphisms (SNPs) are known to influence the response rate to biologic agents, with up to one third of patients not responding to treatment and requiring another agent [29]. For instance, the HLA-Cw6 allele, a predictive marker for the response to the IL12/23-targeting drug UST, and SNPs related to increased IFN-γ levels, have been identified as key factors affecting the therapeutic outcome with UST [30]. The association between genetic variants and therapeutic outcomes indicates that these genetic polymorphisms might not only determine treatment efficacy but also predispose to adverse events. For example, multiple gene polymorphisms, including variants of the IL-23R, have been associated with paradoxical psoriasiform reactions in patients treated with anti-TNF agents [31]. Although specific research on UST-related SNPSs is lacking, given the implication of IL-23R, it is conceivable that specific SNPs may alter the receptor’s functionality or its expression levels, potentially amplifying or diminishing the therapeutic effect of UST, thereby heightening the risk for paradoxical reactions.

One could hypothesize that the interplay of these genetic variations with the drug’s pharmacodynamics could be at the heart of such paradoxical reactions. Furthermore, the role of specific SNPs like IL-12B and IL-23R in psoriasis predisposition, and their influence on cytokine synthesis and T-cell differentiation, can provide a genetic framework that, when altered, may shift the therapeutic response spectrum of UST [32]. This concept is not exclusive to UST. A classic example in pharmacogenomics is the antiretroviral drug abacavir, where the presence of the HLA-B*57:01 allele can predispose patients to a hypersensitivity reaction [33]. Consequently, while the selective inhibition of cytokines remains a viable mechanistic contributor to paradoxical psoriasis, the potential influence of genetic polymorphisms within genes relevant to the drug’s mechanism of action or to the disease process itself cannot be overlooked. This concept is also practically illustrated in our review, where Suh et al. and Safa et al. present a case (Patients #4 and #11) where infliximab-induced paradoxical psoriasis was further exacerbated by UST therapy [20,27]. The authors speculated that genetic polymorphisms were present, which influenced the response to multiple biologic agents, which had differing mechanisms of action.

However, optimal management strategies require further research, and integrating pharmacogenomic approaches into clinical practice might be beneficial for personalized patient response profiles to biologic agents.

No conclusions can be drawn regarding risk factors due to the limited number of cases and the heterogeneity of the patients. However, it appears that paradoxical psoriasis can occur in individuals with no previous history of psoriasis, suggesting that the presence of psoriasis is not a prerequisite for this phenomenon. Clinicians should thus be vigilant about this risk in patients treated with UST, irrespective of their psoriasis history. Moreover, prior exposure to biologics significantly modifies drug survival for biologics. In our cohort, the majority had previous biologic treatments, aligning with findings that previous biologic exposure increases the year-by-year discontinuation probability of UST by up to 15% [34]. In our review, four patients took UST for longer than one year and three of them had prior exposure to biologics. Regular monitoring for signs of psoriasis should be considered, and if paradoxical psoriasis develops, various management strategies can be employed, including the discontinuation of UST, switch to another biologic agent, and the addition of topical corticosteroids or intensification of therapy.

However, optimal management strategies require further research, and integrating pharmacogenomic approaches into clinical practice might be beneficial for personalized patient response profiles to biologic agents.

In conclusion, while UST is effective in treating conditions like psoriasis and Crohn’s disease, the potential for paradoxical psoriasis should be acknowledged. Most cases resolve upon UST discontinuation and switching to another biologic agent. However, some cases respond well to continued UST treatment with added topical corticosteroids or intensified therapy. Further research is needed to understand the risk factors and develop optimal management strategies for UST-induced paradoxical psoriasis.

## 5. Conclusions

While the occurrence of paradoxical psoriasis induced by ustekinumab (UST) is rare, it represents a significant clinical challenge that merits deeper investigation. Our comprehensive review of the available case reports, which includes a case from our clinic, emphasizes the importance of clinician awareness regarding the potential onset of paradoxical psoriasis in patients undergoing UST therapy. The management of this condition demands a tailored approach, taking into account the severity of the paradoxical psoriasis, the patient’s overall health status, and the efficacy of UST in treating the primary illness. In the majority of cases we reviewed, discontinuing UST and switching to another biologic agent resulted in the resolution of paradoxical psoriasis. However, there were instances where the continuation of UST with the addition of topical corticosteroids or intensification of therapy was also effective. This variability in response highlights the need for further research to unravel the risk factors and underlying mechanisms, and develop optimal management strategies for UST-induced paradoxical psoriasis. Advancing our knowledge in this area is crucial not only for understanding the complex pathogenesis of psoriasis but also for enabling clinicians to provide the best possible care to their patients.

## Figures and Tables

**Table 1:** Summary of case reports identified in the literature with PP induced by UST treatment.

Author and Year [Reference]	Patient ID	Age	Sex	Previous Psoriasis History	Ustekinumab Treatment Duration	Dose of UST	How Long after UST Did Lesions Appear	Type of Paradoxical Psoriasis	Location of Paradoxical Psoriasis	Severity (PASI Score)	How Was Paradoxical Psoriasis Flare Controlled	Comorbidities	Therapy Prior to UST	Time to Resolution	Management Strategy (Discontinuation, Switching to Other Agent, Etc.)
Our case	1	39	Female		Three years (from 2020 to 2023)		Three years	Psoriasiform (biopsy-proven)	Hands, elbows, and scalp		Topical steroids	Crohn’s Disease (CD)	Infliximab, adalimumab		Continued UST with topical treatment
Barahimi et al., 2021 [18]	2	51	Male	No history of psoriasis	Three years	90 mg	Three flares separated lasting several weeks; one flare per year	Subcorneal pustular dermatosis	Hands, body, face, extremities, and scalp	10–15 (moderate)	Topical corticosteroids; second flare managed with discontinuation	Crohn’s disease and enteropathic arthritis	Adalimumab, infliximab, and methotrexate	Three weeks	Discontinuation of UST during flares and restarting therapy once flares resolved
Benzaquen et al., 2018 [19]	3	58	Female	No history of psoriasis	Three weeks	390 mg	Three weeks	Paradoxical Palmoplantar Pustular Psoriasis	Right hand palm	0–5 (mild)	Discontinuation, golimumab	Asthma, corticosteroid-induced osteoporosis, arthritis, Crohn’s disease, spondyloarthropathy	Systemic corticosteroids, mesalazine, azathioprine, adalimumab, and infliximab	15 days	Discontinuation of UST, switched to golimumab
Suh et al., 2018 [20]	4	30	Male	6-year history of psoriasis	Two weeks	45 mg	One week after the first injection	Possibly pustular psoriasis, but not definitively diagnosed	Trunk, lower extremities, scalp, palms, and soles	Not available	Discontinuation, oral cyclosporin and a topical agent	Not available	Previously treated with infliximab phototherapy, acitretin, and cyclosporine.	One week	Discontinuation of UST and initiation of treatment with oral cyclosporin and a topical agent
Darwin et al., 2018 [21]	5	56	Female	Palmoplantar psoriasis	15 months	90 mg monthly	15 months	Inverse psoriasis	Inter-gluteal cleft and genital area	0–5 (mild)	Tacrolimus cream daily and pulsed clobetasol cream as necessary on weekends	Crohn’s disease, rheumatoid arthritis	Infliximab, adalimumab, thalidomide, and hydroxychloroquine	Not provided	Medications were not halted as her Crohn’s disease was well controlled under the current medication regimen
Lee et al., 2017 [22]	6	24	Male	7-year history of psoriasis vulgaris	12 weeks	45 mg	After the third injection	Plaque psoriasis	Face, trunk, and extremities	10–15 (moderate)	Systemic steroid and NBUVB phototherapy	None	NBUVB phototherapy, acitretin, methotrexate, and cyclosporine	Two weeks	Addition of systemic steroid and NBUVB phototherapy, but patient discontinued treatments after 2 weeks and was lost to follow-up
Hay et al., 2014 [23]	7	47	Male	15-year history of plaque psoriasis	One month	45 mg	Before the second dose, one month later	Pustular psoriasis	60% of body surface area	>15 (severe)	Discontinuation and adalimumab	Psoriatic arthropathy	Methoxetrate, acitretin, ciclosporin, and narrowband ultraviolet B phototherapy	Two weeks	Discontinuation of UST, switching to adalimumab
Caca-Biljanovska et al., 2013 [24]	8	34	Female	Severe plaque psoriasis since the age of 10	>18 months	45 mg	Week 10, after two injections	Pustular psoriasis	Trunk and limbs	>15 (severe)	Topical corticosteroids and intensification of UST	NA	Topical corticosteroids, psoralen plus ultraviolet A irradiation (PUVA), and acitretin	Four weeks	Continued UST therapy
Wenk et al., 2012 [25]	9	37	Female	10-year history of plaque psoriasis	12 weeks, injection at day 0, week 4, and week 12	45-mg	Four days after first injection and subsequently after second and third injection	Pustular psoriasis	Trunk and limbs	>15 (severe)	Discontinuation and systemic and topical steroids	Psoriatic arthritis	Infliximab, adalimumab, cyclosporine, acitretin, narrow band ultraviolet B irradiation, etanercept, methotrexate, systemic steroid treatment, topical steroids, and acitretin	Not specified	Discontinuation of UST and switch to acitretin
Gregoriou et al., 2011 [26]	10	54	Female	5-year history of plaque psoriasis	NA	45 mg	Two days after first injection	Pustular psoriasis	Trunk and both upper and lower extremities	>15 (severe)	Discontinuation of UST and replacement with methotrexate 25 mg per week with folic acid supplementation	Diabetes type II, hypertension, and hypercholesterolemia	Metformin 500 mg BID for diabetes type II, ramipril 2.5 mg QD for hypertension, and simvastatin 20 mg QD for hypercholesterolemia, topical corticosteroids, calcipotriol, and a calcipotriol-betamethasone dipropionate two compound formulation for 3 years	Two months	Discontinuation of UST and replacement with methotrexate 25 mg per week with folic acid supplementation
Safa et al., 2011 [27]	11	35	Female	No personal history but had a family history	Started at weeks 0 and 4	45 mg	After six weeks and two injections	Exacerbation of Infliximab-induced palmoplantar psoriasis	Palms and soles	Not specified	Improved with oral methotrexate after discontinuation of ustekinumab	Ankylosing spondylitis	Nonsteroidal anti-inflammatory drugs, adalimumab, etanercept, and infliximab	Two months	Discontinuation of ustekinumab

## Data Availability

The data from the literature review is publicly available. The data regarding the case report is available from the corresponding author upon reasonable request.
