# Correction: Olteanu et al. Paradoxical Psoriasis Induced by Ustekinumab: A Comprehensive Review and Case Report. *Medicina* 2024, *60*, 106

**DOI:** 10.3390/medicina61020185

**Published:** 2025-01-22

**Authors:** Andrei Ovidiu Olteanu, Artsiom Klimko, Ioana Tieranu, Olguta Anca Orzan, Cristian Valentin Toma, Elena Mirela Ionescu, Carmen Monica Preda, Cristian George Tieranu

**Affiliations:** 1 Department of Gastroenterology, “Carol Davila” University of Medicine and Pharmacy, 020021 Bucharest, Romania; ovidiu-andrei.olteanu@drd.umfcd.ro (A.O.O.); mirela.ionescu@umfcd.ro (E.M.I.); carmen.preda@umfcd.ro (C.M.P.); cristian.tieranu@umfcd.ro (C.G.T.); 2 Department of Gastroenterology, Elias Emergency University Hospital, 011461 Bucharest, Romania; 3 Laboratory of Molecular Neuro-Oncology, Department of Neurology, University Hospital Zurich, 8091 Zürich, Switzerland; artsiom.klimko@usz.ch; 4 Department of Pediatrics, “Maria Sklodowska Curie” Clinical Emergency Hospital for Children, 077120 Bucharest, Romania; dr.cindeaioana@yahoo.ro; 5 Department of Dermatology, “Carol Davila” University of Medicine and Pharmacy, 020021 Bucharest, Romania; 6 Department of Dermatology, Elias Emergency University Hospital, 011461 Bucharest, Romania; 7 Department of Innovation and e-Health, “Carol Davila” University of Medicine and Pharmacy, 020021 Bucharest, Romania; cristian.toma@umfcd.ro; 8 Department of Gastroenterology, Fundeni Clinical Institute, 022328 Bucharest, Romania

## Author Order

In the original publication [1], there is an error in the order of the authors, and the author’s affiliation also needs to be changed accordingly. The correct version is as follows:

**Andrei Ovidiu Olteanu ^1,2,†^, Artsiom Klimko ^3,^** ^†^, Ioana Tieranu ^4^, Olguta Anca Orzan ^5,6,^*, Cristian Valentin Toma ^7^, Elena Mirela Ionescu ^1,2^, Carmen Monica Preda ^1,8^ and Cristian George Tieranu ^1,2^

^1^  Department of Gastroenterology, “Carol Davila” University of Medicine and Pharmacy, 020021 Bucharest, Romania; ovidiu-andrei.olteanu@drd.umfcd.ro (A.O.O.); mirela.ionescu@umfcd.ro (E.M.I.); carmen.preda@umfcd.ro (C.M.P.); cristian.tieranu@umfcd.ro (C.G.T.)
^2^  Department of Gastroenterology, Elias Emergency University Hospital, 011461 Bucharest, Romania
^3^  Laboratory of Molecular Neuro-Oncology, Department of Neurology, University Hospital Zurich, 8091 Zürich, Switzerland; artsiom.klimko@usz.ch

The inovation of affiliation 7 should be innovation. Other details remain unchanged.

The authors state that the scientific conclusions are unaffected. This correction was approved by the Academic Editor. The original publication has also been updated.

## References

[1] Olteanu A.O., Klimko A., Tieranu I., Orzan O.A., Toma C.V., Ionescu E.M., Preda C.M., Tieranu C.G. (2024). Paradoxical Psoriasis Induced by Ustekinumab: A Comprehensive Review and Case Report. Medicina.

